# Identification and Characterization of Serum microRNAs as Biomarkers for Human Disc Degeneration: An RNA Sequencing Analysis

**DOI:** 10.3390/diagnostics10121063

**Published:** 2020-12-08

**Authors:** Shangbin Cui, Zhiyu Zhou, Xizhe Liu, Robert Geoff Richards, Mauro Alini, Songlin Peng, Shaoyu Liu, Xuenong Zou, Zhen Li, Sibylle Grad

**Affiliations:** 1 Guangdong Provincial Key Laboratory of Orthopedics and Traumatology, The First Affiliated Hospital of Sun Yat-sen University, Guangzhou 510000, China; shangbin.cui@aofoundation.org (S.C.); zzy990802@126.com (Z.Z.); gzxzhliu@gmail.com (X.L.); geoff.richards@aofoundation.org (R.G.R.); gzsyliu@tom.com (S.L.); zouxuen@mail.sysu.edu.cn (X.Z.); 2 AO Research Institute Davos, 7270 Davos, Switzerland; mauro.alini@aofoundation.org (M.A.); zhen.li@aofoundation.org (Z.L.); 3 The Seventh Affiliated Hospital of Sun Yat-sen University, Shenzhen 518107, China; 4 Department of Spine Surgery, Shenzhen People’s Hospital, Shenzhen 518020, China; songlin824@gmail.com

**Keywords:** circulating microRNA, intervertebral disc, degeneration, disc herniation, biomarker

## Abstract

Circulating microRNAs (miRNAs) have been associated with various degenerative diseases, including intervertebral disc (IVD) degeneration. Lumbar disc herniation (LDH) often occurs in young patients, although the underlying mechanisms are poorly understood. The aim of this work was to generate RNA deep sequencing data of peripheral blood samples from patients suffering from LDH, identify circulating miRNAs, and analyze them using bioinformatics applications. Serum was collected from 10 patients with LDH (Disc Degeneration Group); 10 patients without LDH served as the Control Group. RNA sequencing analysis identified 73 differential circulating miRNAs (*p* < 0.05) between the Disc Degeneration Group and Control Group. Gene ontology enrichment analysis (*p* < 0.05) showed that these differentially expressed miRNAs were associated with extracellular matrix, damage reactions, inflammatory reactions, and regulation of apoptosis. Kyoto Encyclopedia of Genes and Genomes analysis showed that the differentially expressed genes were involved in diverse signaling pathways. The profile of miR-766-3p, miR-6749-3p, and miR-4632-5p serum miRNAs was significantly enriched (*p* < 0.05) in multiple pathways associated with IVD degeneration. miR-766-3p, miR-6749-3p, and miR-4632-5p signature from serum may serve as a noninvasive diagnostic biomarker for LHD manifestation of IVD degeneration. Furthermore, several dysregulated miRNAs may be involved in the pathogenesis of IVD degeneration. Further study is needed to confirm the functional role of the identified miRNAs.

## 1. Introduction

Low back pain (LBP) can cause severe disabilities and is associated with significant personal, social, and economic burden. Reports from the Global Burden of Disease Study 2015 showed that 7.3% of the global population (540 million) had activity-limiting LBP in the global point prevalence survey [1]. Epidemiological and clinical studies have demonstrated that LBP had a critical relationship with lumbar intervertebral disc (IVD) degeneration. While the etiology of LBP is multifactorial, it was reported to be associated with IVD degeneration in up to 40% of cases [2]. Approximately 40% of people < 30 years old, and >90% of people > 55 years, display moderate-to-serious degrees of IVD degeneration [3]. The etiology of IVD degeneration implies both genetic and environmental factors. However, environmental factors such as vibration loading at work, heavy mechanical overload, physical activities, and smoking are weakly accountable for the development and progression of IVD degeneration [4]. In contrast, genetic heredity is considered a predominant risk factor for premature IVD degeneration and is believed to be causative in more than 70% of cases [5].

Lumbar disc herniation (LDH) is one of the most prominent manifestations of IVD degeneration. Up to now, the diagnosis of LDH is merely based on clinical observation and magnetic resonance imaging (MRI). A better understanding of the molecular events essential in the pathogenesis of IVD degeneration and associated LDH could represent a critical improvement in the development of new diagnostic tools. Gene expression profiling has widely been utilized to analyze gene expression regulation, to understand how genes are related to certain biological functions, and to clarify the pathogenic mechanisms of diseases. Nowadays, with the advancement of new sequencing methods, large parallel sequencing of RNA is widely used for gene expression profiling, non-coding transcripts identification, and alternative splicing variants detection [6].

MicroRNAs (miRNAs) are a class of small non-coding RNAs that regulate gene expression and play important roles in essential physiological and pathological processes. It has been found that although miRNAs represent just 1–3% of the human genome, they have the ability to regulate around 30% of the protein-encoding genes in humans [7,8,9]. Studies have shown that miRNAs are dysregulated in many diseases, including IVD disorders [10,11], and can be released into circulation [12,13,14]. Therefore, miRNAs show great potential in biomarker discovery and diagnostic screening. Circulating miRNAs are present in blood serum or plasma, which are easier and less invasive to collect than traditional tissue biopsies. MiRNA contains few nucleotides and is relatively stable due to interactions with protective mechanisms, including exosomes, extracellular vesicles, and protein complexes [15,16].

In the present study, patients with IVD degeneration and manifest LDH were enrolled to reduce the heterogeneity among IVD degeneration phenotypes. The aim was to generate circulating miRNA profiles and compare them with the miRNA profiles of patients without IVD degeneration. From the differential expression profiles, the associated biological processes and the occurrence of potential biomarkers of IVD degeneration with LDH were evaluated.

## 2. Materials and Methods 

### 2.1. Patients 

Twenty patients were enrolled in this study, including 10 patients diagnosed as having LDH (Disc Degeneration Group, DD Group, 53.1 ± 24.2 years old) and 10 patients diagnosed as having other spinal diseases without IVD degeneration (Control Group, 25.1 ± 10.2 years old). Patients with known chronic diseases such as diabetes, hypertension, arthritis, and rheumatism, were excluded from the study. MRI data were used to confirm the manifestation of LDH in the DD Group and exclude the occurrence of IVD degeneration in the Control Group. The Pfirrmann Grading System was used to assess IVD degeneration grade. In the DD group, there were 3 patients assessed as Grade IV and 7 patients assessed as Grade V. In the control group, all patients were assessed as Grade I. Written informed consent was obtained from every patient. This study was approved by the ethics committee of the first affiliated hospital of Sun Yat-sen University (29 March 2018, Guangzhou, China; nr. (2018)-053). The clinical and demographic characteristics of the study participants are shown in Table 1.

### 2.2. Collection of Serum from Peripheral Blood

Peripheral blood samples were collected from the patients in the first affiliated hospital of Sun Yat-sen University prior to surgery between 7:30 a.m. and 10:30 a.m. to limit the effect of circadian variation. From each patient, 10 mL whole blood was withdrawn and put into a vacutainer tube without an anticoagulation agent. After collection, the blood was allowed to clot by keeping it undisturbed at room temperature for 20 min. Samples were then centrifuged at 4 °C for 10 min (2000× *g*) to remove residual cells and debris. The supernatant was collected and transferred to a new centrifuge tube. The tubes containing the serum were stored in liquid nitrogen.

### 2.3. RNA Extraction

Total RNA was extracted using TRIzol^®^ reagent (Invitrogen, Carlsbad, CA, USA) as previously described [17,18]. Plasma was homogenized with TRIzol^®^ reagent, followed by the addition of chloroform. Each sample was vortexed for 30 s and incubated at room temperature for 5 min. Then, the samples were centrifuged at 12,000× *g* for 15 min at 4 °C. The upper aqueous phase was transferred to a new tube. A mixture of glycogen (Invitrogen, San Diego, CA, USA) and tRNA (Sigma-Aldrich, St. Louis, MO, USA) for RNA precipitation was added to the aqueous phase before being mixed with sodium acetate and isopropanol. The tube was vortexed for 30 s and incubated at −80 °C for 1 h. The tube was then centrifuged at 20,000× g for 30 min at 4 °C. After centrifugation, the supernatant was discarded, and the RNA pellet was dissolved in 30 μL of RNase-free water [17]. The Agilent 2100 Bioanalyzer (Agilent, Santa Clara, CA, USA) was used to analyze RNA integrity and concentration, and the NanoDrop 2000 spectrophotometer (Thermofisher Scientific, Waltham, MA, USA) was used to detect inorganic ions or polycarbonate contamination. For all samples, the total RNA amount was >5 μg, and the RNA concentration was >200 ng/μL.

### 2.4. Filtering of Small RNAs

From each sample, 200 ng–1 μg of RNA was separated into segments of different size by polyacrylamide gel electrophoresis (PAGE), and the 18–30 nt segment was selected (14–30 ssRNA ladder marker, TAKARA). For adaptor ligation, the connection 3′adaptor system was prepared (reaction condition: 70 °C for 2 min; 25 °C for 2 h); secondly, RT-primer was added (reaction condition: 65 °C for 15 min; ramp to 4°C at a rate of 0.3 °C/sec); thirdly, the 5′adaptor mix system was added (reaction condition: 70 °C for 2 min; 25 °C for 1 h). 

### 2.5. Reverse Transcription Polymerase Chain Reaction (RT-PCR)

First strand cDNA synthesis was performed using Super Script II (Invitrogen) reverse transcription reagents (reaction condition: 42 °C for 1 h; 70 °C for 15 min). A few rounds of PCR amplification with PCR primer cocktail and PCR Master Mix were carried out to enrich the cDNA fragments (reaction condition: 95 °C for 3 min; 15–18 cycles of (98 °C for 20 s, 56 °C for 15 s, 72 °C for 15 s); 72 °C for 10 min; 4 °C hold). 

### 2.6. Purification of PCR Products, Circularization, Library Validation, and Sequencing

The PCR products were purified with polyacrylamide gel electrophoresis (PAGE) and the recycled products were dissolved in ethylene dibromide solution. The double stranded PCR products were then heat denatured and circularized by the splint oligo sequence. The single stranded circle DNAs were designed as the final library. The Agilent Technologies 2100 Bioanalyzer was used to validate the library. The library was amplified with phi29 to make DNA nanoballs (DNBs) that have more than 300 duplicates of one molecule. The DNBs were loaded into the patterned nanoarray and single end 50 bases reads were produced by the combinatorial probe-anchor synthesis (cPAS) method.

### 2.7. Data Processing and Differentially Expressed Gene (DEG) Screening

The relationship between miRNA and the target gene was resolved depending on the original expression profiling, and log_2_-transformed deep sequencing data were used to screen the differentially expressed genes (DEGs) between groups by linear models of the LIMMA package in R [12]. The method of Benjamini and Hochberg was utilized to calculate false discovery rates (FDRs) [19]. FDR-corrected *p* < 0.05 and |log(fold change)| > 1 were set as the thresholds for screening miRNAs.

### 2.8. Differentially Expressed Analysis of miRNA and the Target Genes

The current investigation utilized three popular databases, namely MiRBas (Available online: www.mirbase.org, accessed on 1 may 2020), TargetScan (Available online: www.targetscan.org, accessed on 1 May 2020), and MiRanda (Available online: www.microrna.org, accessed on 1 May 2020) to anticipate the target genes which were related to significantly differentially expressed miRNAs. Target genes, which were overlapping in all three databases, were chosen for further function annotation analysis. To additionally investigate the association between blood serum miRNA and target genes in the development of IVD degeneration, the target genes were further analyzed by the protein–protein interaction (PPI) network.

### 2.9. Establishment and Analysis of miRNA Regulated Gene Networks

The online Search Tool for the Retrieval of Interacting Genes (Available online: STRING; http://string-db.org, accessed on 1 May 2020) is a tool that can cluster networks on request and update on-screen previews of structural information including homology models and extensive data updates. To investigate all functional interactions among proteins, STRING was utilized to determine the relationship between the differentially expressed miRNAs and target genes. A confidence score was used to characterize the connection between miRNA and genes by experimental measurements and computational prediction techniques. The confidence score > 0.4 was set as statistically significant. The Cytoscape tool was used for the interaction network’s visualization. The Biological Networks Gene Ontology Tool (Available online: BiNGO; http://www.psb.ugent.be/cbd/papers/BiNGO, accessed on 1 May 2020), as a Cytoscape plugin, is an open-source Java tool to identify significantly overrepresented target gene ontology terms in a set of genes [20].

### 2.10. Gene Ontology (GO) and Kyoto Encyclopedia of Genes and Genomes (KEGG) Analysis

Differentially expressed gene enrichment was analyzed from GO by GOATOOLS and included molecular function, cell components, and biological processes. The Database for Annotation, Visualization, and Integrated Discovery (DAVID) was used for GO functional enrichment analysis with a threshold of *p* < 0.05 [21]. KEGG (Available online: www.genome.jp/kegg.pathway.html, accessed on 1 May 2020) and DAVID (Available online: https://david.ncifcrf.gov/, accessed on 1 May 2020), which offer an exhaustive set of functional annotation tools to investigate the genes, were utilized for pathway analysis. 

### 2.11. Statistical Analysis

Data were analyzed using SPSS version 19.0 (IBM SPSS Inc., Chicago, USA). The differences between groups were assessed using the *t*-test. A *p*-value < 0.05 was considered statistically significant.

## 3. Results

### 3.1. Quality Analysis

The quality of the data was initially assessed as shown in Table 2. The bases with a Phred quality score > 20 accounted for about 97% of the total bases, indicating that the original sequencing data were of good quality and could be used for subsequent analysis.

### 3.2. Differentially Expressed miRNA and Target DEG Analysis

**Figure 1:**
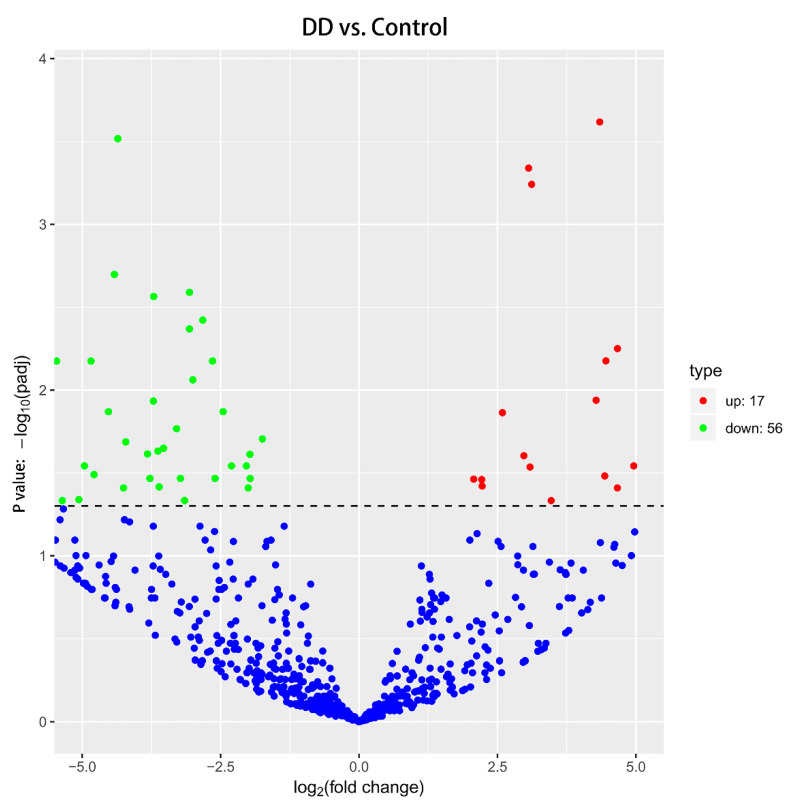
Volcano map of the differentially expressed miRNAs. The left part shows the downregulated miRNAs, and the right part indicates the upregulated miRNAs in the DD Group versus the Control Group (y-axis represents *p* value; x-axis represents log2 fold change).

Using R software version 3.1.1 (from bioconductor.org/packages/release/bioc/html/biomaRt.html), 73 differentially expressed miRNAs were identified between the two study groups by the screening criteria of multiple expression of differentially expressed genes (fold change > 1 and *p* < 0.05). There were 17 miRNAs upregulated and 56 miRNAs downregulated in the serum from patients with lumbar disc herniation (DD Group) compared with the Control Group (Figure 1); 570,867 target genes were obtained based on their relationship between the miRNAs and related genes in the database. The dysregulated miRNAs are shown in Table 3.

### 3.3. Gene Ontology (GO) Functional Enrichment Analysis

**Figure 2:**
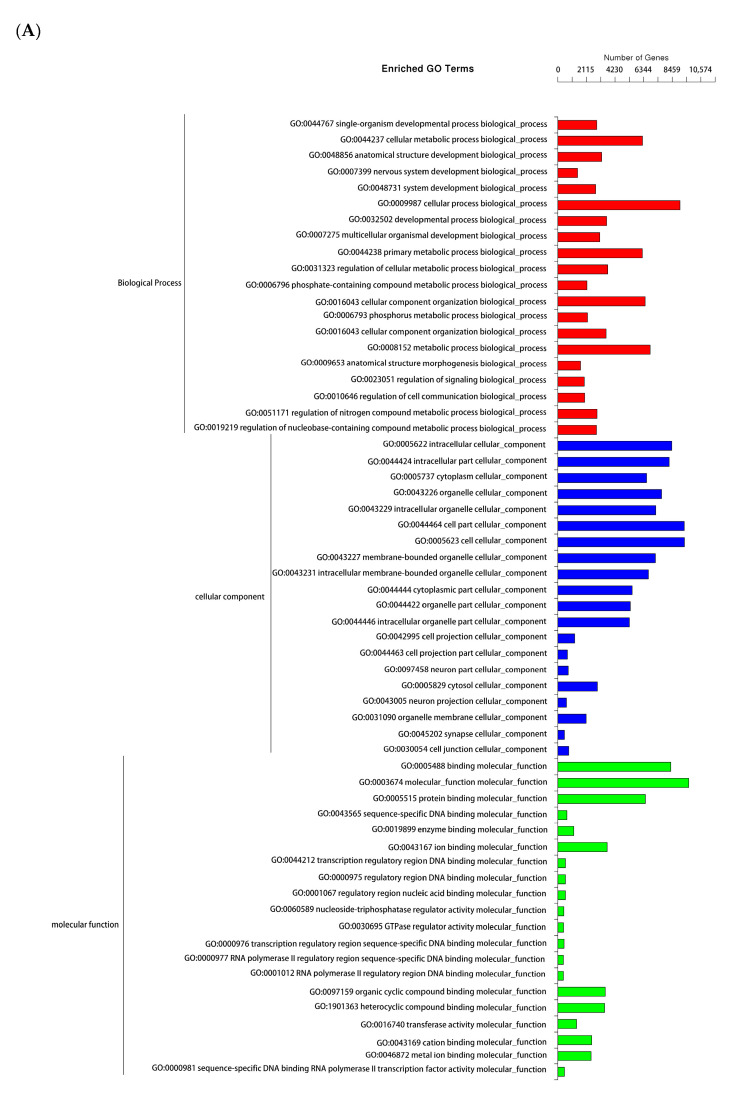

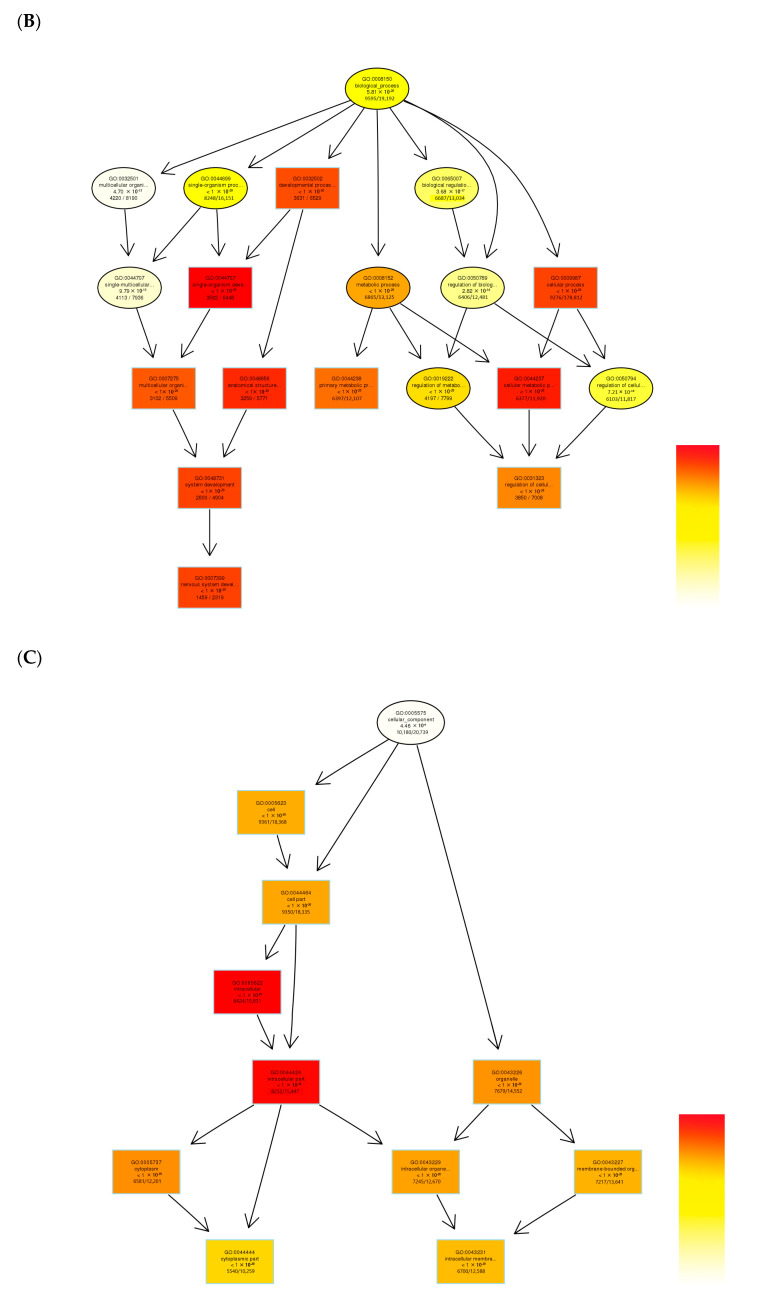

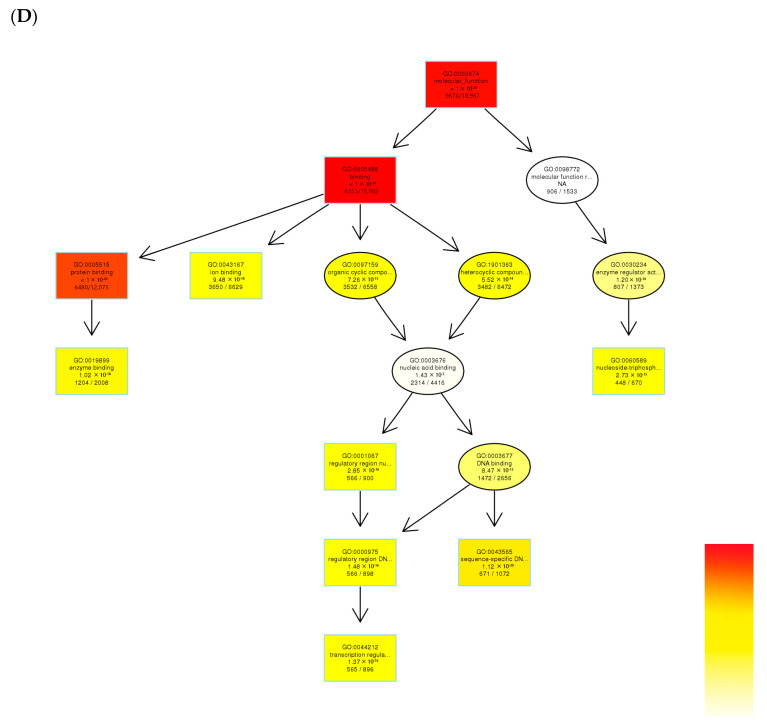
Enriched gene ontology (GO) terms and directed acyclic graphs (DAG). (**A**) The first 20 GO terms. (**B**–**D**) Directed acyclic graph showing the GO enrichment analysis results of the candidate target gene, with branches representing the functional range by the inclusion relationship. The top 10 GO terms were selected as the main nodes of the directed acyclic graphs to map the candidate target gene DAG of the biological process, molecular function, and cellular component, respectively. The depth of the color represents the enrichment degree.

All genes that were targeted by the differentially expressed miRNAs were mapped to terms in the GO database. GO analysis of biological processes showed that the differentially expressed genes were enriched in three GO terms, including cellular component, biological process, and molecular function (Figure 2A). The first 10 GO terms in the results were selected as the main nodes of the directed acyclic graphs and the related GO term was displayed together, showing the inclusion relationship (Figure 2B–D). 

### 3.4. Enrichment Analysis

**Figure 3:**
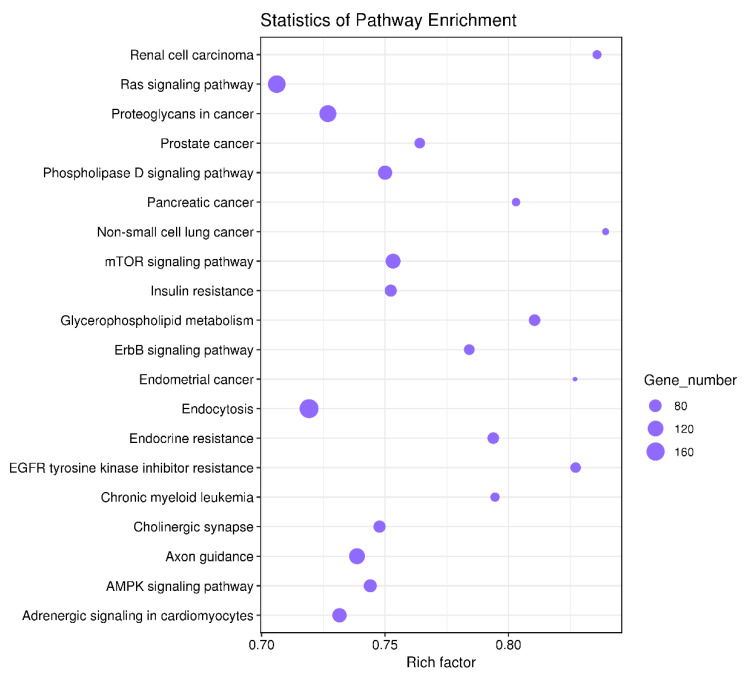
The top 20 signaling pathways regulated by the significantly differentially expressed miRNAs. Rich factor is defined as the ratio of the number of target genes to the absolute number of genes in the pathway. The larger the factor represented, the greater the enrichment degree. The area of the spot represents the number of target genes included in the pathway.

The genes targeted by the significantly differentially expressed miRNAs were enriched in different signaling pathways as identified by KEGG analysis. The top 20 signaling pathways are presented in Figure 3. 

### 3.5. miRNA Target Gene Regulation Association and miRNA-DEG Regulation Network Construction

From the TargetScan database, target genes of 10 upregulated and downregulated differentially expressed miRNAs were obtained. The abnormally expressed miRNAs were then screened by using conservative property at 3′UTR and context score. Among them, the genes showing most relevance in the pathways related to IVD degeneration were selected according to the literature, including endocytosis, apoptosis, axon guidance, vascular endothelial growth factor (VEGF), regulation of cytoskeleton, cell adhesion molecular, focal adhesion, extracellular matrix (ECM)-receptor interaction, phosphoinositide 3-kinase (PI3K)/Akt, mTOR, and NF-κB pathways [22,23,24,25,26]. The top miRNAs which had the highest confidence score were identified and shown in Table 4. The upregulated miR-766-3p and miR-6749-3p play a role in most of the pathways. Additionally, the downregulated miR-4632-5p plays a role in most of the pathways.

**Figure 4:**
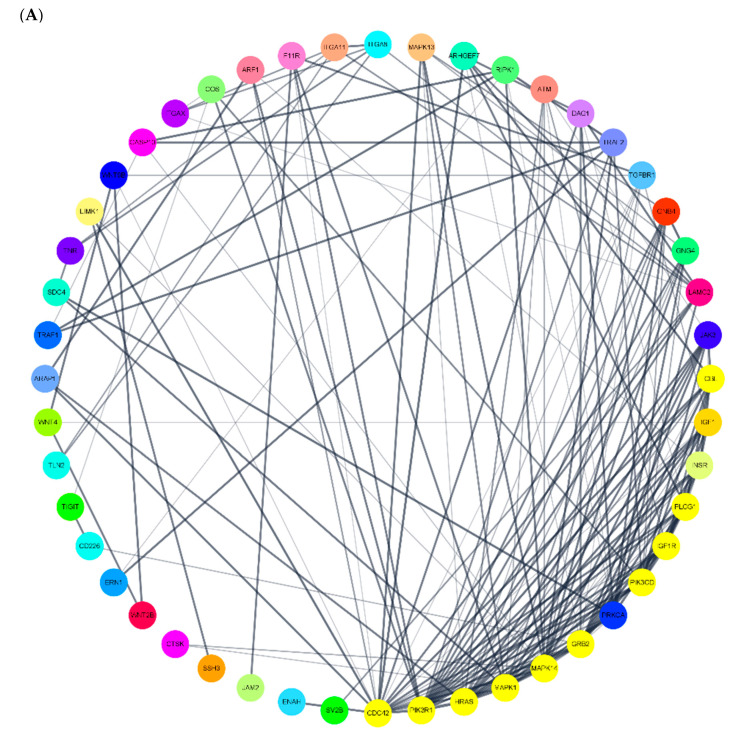

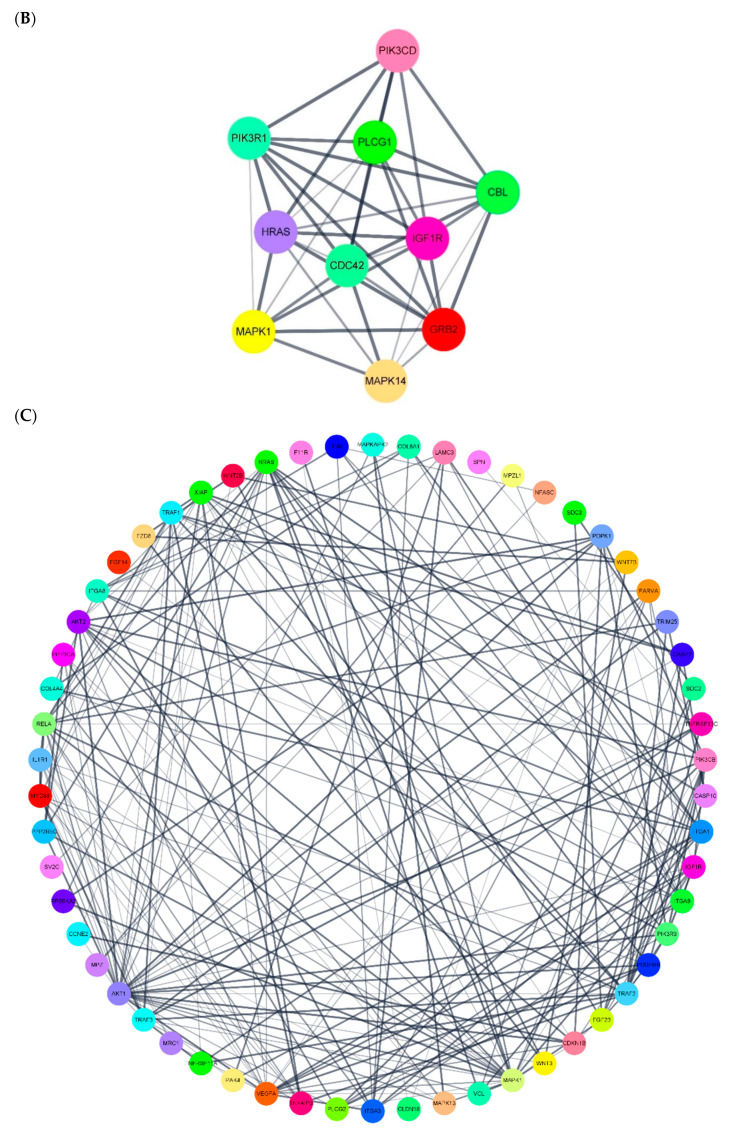

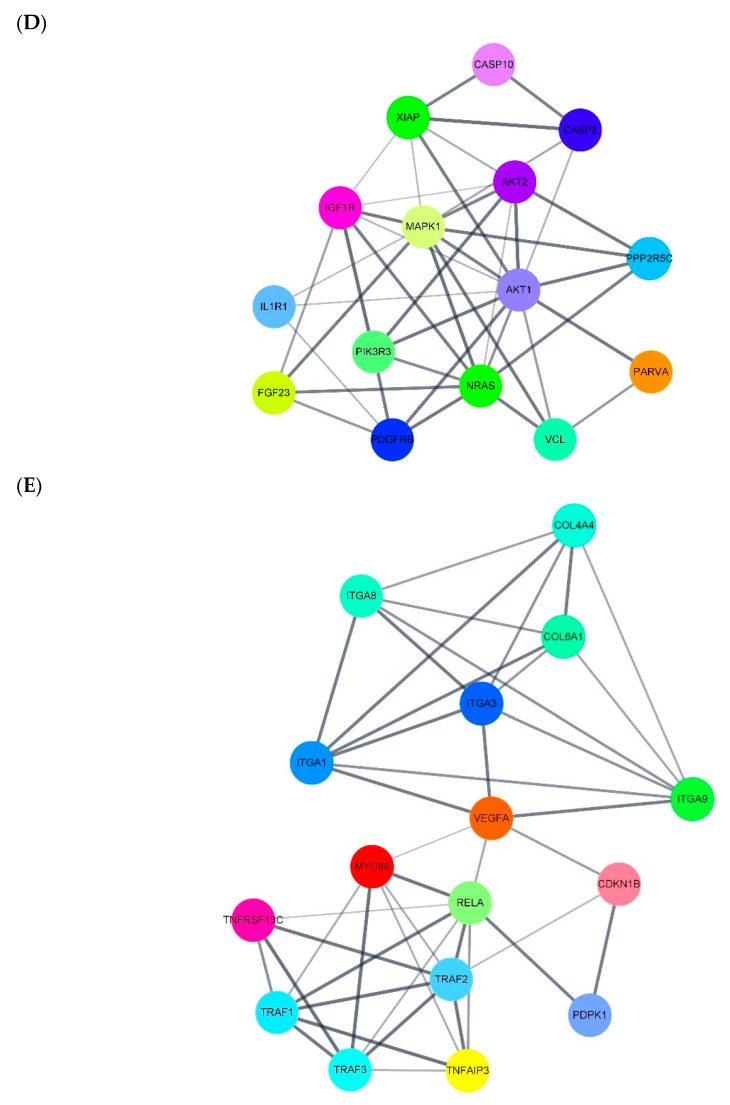
Identification of hub genes from the PPI network by using the MCODE algorithm. (**A**,**C**) represent the PPI networks of upregulated genes and downregulated genes, respectively. (**B**) (clustering score = 8) is the PPI network of hub genes extracted from (**A**). (**D**) (clustering score = 6) and (**E**) (clustering score = 5.73) are the PPI networks of hub genes extracted from (**C**). PPI: protein–protein interaction; MCODE: Molecular Complex Detection.

To investigate the regulation networks of the target genes regulated by the miRNAs, the relationship between proteins encoded by target genes was identified by mapping both upregulated and downregulated genes to STRING. The regulation networks are shown in Figure 4. Cluster analysis was performed by using the Molecular Complex Detection Algorithm (MCODE) plugin (version: 1.5.1) in Cytoscape [27,28] for the identification of the clustering modules in the PPI network. Significant modules were identified according to the clustering score with following criteria: “Degree cutoff = 2”, “Node score cutoff = 0.2”, “Haircut = true”, “Fluff = false”, “k core = 2”, and “Max depth = 100”. The clustering modules which had high connectivity degrees and node scores were identified as biologically significant clusters.

## 4. Discussion

An ideal biomarker should be easily assessed with minimally invasive or non-invasive medical procedures and possess high specificity and sensitivity. Human serum or plasma has been a rich source of biomarkers for a long time. Recent studies showed that circulating RNA molecules, such as miRNAs, can be profiled to identify new promising biomarkers for many diseases [10,29]. By surveying human serum for miRNA biomarkers with the new sequencing technology, we found that a significant portion of circulating miRNAs appeared to be dysregulated in patients with LDH. To our knowledge, this is the first study elucidating the global transcriptome of differentially expressed circulating miRNAs between controls and LDH patients by using RNA-seq technology. In the present study, 17 upregulated and 56 downregulated miRNAs were found between the study groups. Furthermore, we demonstrate that IVD degeneration with LDH may be modulated by the dysregulation of several miRNAs. Specifically, upregulated miR-766-3p and miR-6749-3p, and downregulated miR-4632-5p can target multiple genes which are related to IVD degeneration. Furthermore, GO and KEGG pathway analyses uncovered that the differentially expressed miRNAs were widely involved in regulating diverse signaling pathways and cellular processes of human IVD cells, suggesting that they may represent important biomarkers of IVD degeneration. 

Recently, much attention has been paid to the pathogenic mechanisms of IVD degeneration [30,31]. Differentially expressed miRNAs were found in degenerative human IVD tissues and cells. Among these, some miRNAs have been associated with multiple pathological processes during IVD degeneration, including ECM degradation, apoptosis, cell inflammatory responses, and proliferation [10,11]. Emerging evidence has firmly indicated that abnormal miRNA expression may play an important role in IVD degeneration [32].

By comparing spinal cord injury and LDH patients, Zhao et al. identified 25 upregulated miRNAs and 26 downregulated miRNAs in the IVD [33]. Hu et al. investigated the expression patterns of a total of 253 miRNAs in human degenerated nucleus pulposus (NP) cells (from three patients with IVD degeneration) and non-degenerated NP cells (from three controls with scoliosis). The results showed that miR-222, miR-220b, miR-532-3p, miR-640, miR-589, and miR-1286 were confirmed as significantly overexpressed, while miR-30c-1, miR-638, and miR-1275 were validated to be downregulated [23]. Another screening of IVD degeneration-associated miRNAs in patient serum showed that miR-155-5p was significantly downregulated when comparing patients diagnosed with IVD degeneration to healthy individuals (n = 3 each) [34]. Bioinformatic analysis showed that PI3K/Akt, MAPK, and Wnt pathways were most likely controlled by these dysregulated miRNAs [33], which had been proven to be crucial for the pathogenesis of IVD degeneration [35,36,37,38].

In the present study, functional annotation of dysregulated genes targeted by miRNAs was conducted by GO and KEGG pathway enrichment analysis. Interestingly, the top GO BPs which included cellular metabolic process (GO:0044237), cellular component organization (GO:0016043), nervous system development biological process (GO:0007399), regulation of cell communication (GO:0010646), and regulation of signaling (GO:0023051) were correlated with KEGG-enriched pathways which are related to IVD degeneration. These KEGG-enriched pathways included cell death (apoptosis), neurovascular ingrowth (axon guidance, VEGF pathway), cytoskeleton (regulation of cytoskeleton, cell adhesion molecular, focal adhesion, ECM-receptor interaction pathways), and inflammation-related pathways (PI3K/Akt, mTOR and NF-κB pathways).

Cell death is a fundamental biological process. The deregulation of cell death is associated with the etiology and pathogenesis of many, particularly degenerative, diseases [39]. With aging, IVDs gradually degenerate due to a combination of many factors. Human trials and animal studies have documented that cell death, particularly apoptosis and autophagy, significantly contribute to IVD degeneration [39]. Our study suggests that the apoptosis pathways were dysregulated by miR-766-3p, miR-6749-3p, miR-4632-5p, and miR-6165 in manifest LDH. MiR-766-3p has also been regarded as a biomarker for cancer [40,41,42] and post exercise change [43,44]. The expression of miR-766-3p in acute promyelocytic leukemia cells was elevated, while that of BAX, which is a pro-apoptotic protein, was suppressed [45]. Studies also found the expression of miR-766-3p was related to aging [46]. Qian et al. found that downregulation of miR-4632 by platelet-derived growth factor (PDGF)-BB was associated with histone deacetylation through the activation of PDGFR/PI3K/HDAC4 signaling. Their results suggested that miR-4632 plays an important role in the regulation of human pulmonary artery smooth muscle cell apoptosis and proliferation by suppression of cJUN [47]. Moreover, miR-6165 was found to induce apoptosis in human cell lines. Hassanlou et al. showed that miR-6165 overexpression in SW480 cells could significantly downregulate IGF-1R expression and the transcript levels of PI3KR3, PI3KR5, AKT2, AKT3, CCND1, P21, and c-MYC genes. Research on Annexin V showed that miR-6165 overexpression could increase apoptosis and cause a reduction in viability of SW480 cells [48]. 

During IVD degeneration, cytokines are produced within the disc, which can stimulate the ingrowth of nerves and vascular elements that may play a role in the etiology of spinal pain and further degradation of the IVD [49]. Studies also found that NP and annulus fibrosus (AF) of symptomatic degenerated IVDs are often accompanied by aberrant neurovascular ingrowth [50,51]. We further analyzed the miRNA-targeted genes which were enriched in VEGF and axon guidance pathways. The bioinformatic analysis results showed that miR-766-3p, miR-6749-3p, and miR-6165 played an important role in these pathways. Hassanlou et al. also reported that the overexpression of miR-6165 could downregulate ABLIM-1, PVRL1, and PDK1 target genes, thereby affecting cell cycle progression and inhibiting NT2 neural cell differentiation [52].

Cell–matrix adhesion has essential roles in a number of important biological processes, including cell motility, proliferation, differentiation, the regulation of gene expression, and cell survival; at contact points between the cell and ECM, specialized structures termed focal adhesions are formed [53]. The response of cells to loading, and the specific mechanotransduction pathways mediating responses to load, are dependent on cell morphology, cell–cell interactions, and cell–ECM interactions. In our study, the cell adhesion molecular pathway, focal adhesion pathway, and ECM–receptor interaction pathway were also found dysregulated by miR-766-3p, miR-6749-3p, miR-4452, miR-1303, miR-6165, and miR-5006-5p. Previous reports have suggested that multiple mechanotransduction pathways were associated with IVD degeneration, such as focal adhesion, adhesion junction pathways, and actin cytoskeleton regulation [54,55]. Gao et al. found that miR-6819-3p was higher in alcohol-associated hepatocellular carcinoma (HCC) compared with non-alcohol-associated HCC tissues, whereby ACTG1, which was enriched in the regulation of the cytoskeleton pathway, was proposed to be the target of miR-6819-3p [56]. A study by Díaz-Prado and co-authors showed that miR-1227 was downregulated in osteoarthritic chondrocytes compared with normal chondrocytes. TGF-β, MAPK, Wnt, and mTOR signaling, focal adhesion, and regulation of actin cytoskeleton pathways were potentially altered by the differentially expressed miRNAs [57].

An inflammatory response is thought to initiate IVD degeneration, and pro-inflammatory molecules, secreted by IVD cells, are considered to mediate the degeneration process [58]. These cytokines trigger a range of pathogenic responses by the disc cells that can promote autophagy, senescence, and apoptosis [59,60]. The resulting imbalance between catabolic and anabolic responses leads to degeneration, as well as herniation and radicular pain [58]. IVD degeneration and herniation may further trigger the recruitment and activation of different immune and inflammatory cells. The current study also found that the targeted genes were enriched in multiple inflammatory pathways, including the PI3K/Akt signaling pathway and mTOR and NF-κB pathways which were targeted by miR-766-3p, miR-6749-3p, miR-5006-5p, miR-6165, miR4632-5p, and miR-27a-3p. The PI3K/Akt pathway was found to be involved in inflammation by interaction with the mTOR pathway [61,62]. This pathway could also be activated by IVD degeneration [35,63,64]. Zhao et al. found that the expression levels of miR-27a-3p were upregulated in a renal ischemia–reperfusion injury model. miR-27a-3p markedly downregulated p-PI3K, p-AKT, Nrf2, and HO-1 Grb2 and upregulated keap1 expression in model groups. In vitro, miR-27a-3p caused oxidative stress via increasing ROS levels and downregulated PI3K/Akt signaling by mimicking hypoxia reoxygenation [65]. Moreover, the present study found that miR-766-3p and miR-3620-3p target genes were enriched in mTOR and NF-κB pathways. Hayakawa et al. found that miR-766-3p had anti-inflammatory effects and verified its biological function in human rheumatoid arthritis fibroblast-like synoviocyte MH7A cells. MiR-766-3p indirectly reduced mineralocorticoid receptor expression and suppressed cytokine-induced NF-κB activation [66]. Wang and co-authors found that miR-3620-3p was upregulated in chronic obstructive pulmonary disease. Those miRNAs were proven to act in inflammation modulation, regulation of proliferation, differentiation, and oxidative stress [67].

The findings of the present study therefore predict that significantly differentially expressed miRNAs were involved in the regulation of these signaling pathways during the phase of IVD degeneration.

## 5. Conclusions

In conclusion, our RNA sequencing profiles were of good quality and accurate. We found distinct highly dysregulated miRNAs in serum, including miR-766-3p, miR-6749-3p, and miR-4632-5p, which could be used as a new combination of specific biomarkers for predictive diagnosis of IVD degeneration and LDH. Moreover, the present work discovered possible target genes related to LDH pathogenesis. These miRNAs may open new insights into the biology of IVD degeneration and pathogenesis of LDH. Further studies will be designed to verify the mechanism of these miRNAs in different related pathways. The downstream target genes, mRNAs, and proteins may be validated in patients suffering from IVD degeneration, whereas miRNAs may be used for early detection, progression monitoring, and treatment guidance. Moreover, advanced therapies could be designed based on the miRNAs to reverse the degeneration of IVD. 

The present study had several limitations, including small sample size, lack of age-matched groups, and nonrandomized design, which make the study a preliminary analysis. The control group was selected from patients without any IVD degeneration by MRI scan, but they might have had other diseases such as fracture or infection which may interfere with the results. Additionally, the results of the analysis from the deep sequencing data were not verified with other measurement techniques. Numerous other diseases could cause variations in serum miRNAs such as obesity, infection, or diabetes, which, along with the extracellular instability of miRNAs, poses a major limitation and challenge in finding LDH-specific serum miRNAs [68]. Further studies on miRNA functions and target gene verification would provide an experimental basis for the diagnosis and treatment of IVD degeneration, which remains an important area for future investigation.

## Figures and Tables

**Table 1:** Clinical information of the patients included in the study. DD 1–10: Disc Degeneration Group, diagnosed as lumbar disc herniation; Control 1–10: Control Group without disc degeneration.

Sample	Gender	Age (Years)	Clinical Diagnosis
DD 1	male	58	Lumbar disc herniation
DD 2	male	83	Lumbar disc herniation
DD 3	female	67	Lumbar disc herniation
DD 4	male	43	Lumbar disc herniation
DD 5	male	32	Lumbar disc herniation
DD 6	male	32	Lumbar disc herniation
DD 7	female	20	Lumbar disc herniation
DD 8	male	27	Lumbar disc herniation
DD 9	male	56	Lumbar disc herniation
DD 10	female	63	Lumbar disc herniation
Control 1	female	15	Idiopathic scoliosis
Control 2	female	26	C2 Hangman fracture
Control 3	female	14	Idiopathic scoliosis
Control 4	male	31	Lumbar spine tuberculosis infection
Control 5	female	49	T3 vertebrae body fracture
Control 6	male	11	Idiopathic scoliosis
Control 7	female	22	Idiopathic scoliosis
Control 8	female	26	Idiopathic scoliosis
Control 9	male	12	Idiopathic scoliosis
Control 10	male	26	Spine hemangioma

**Table 2:** Quality characteristics of the original sequencing data.

Sample	No. of Total Reads	No. of Clean Reads	Q20	Q30	Mapped Known Total Small RNA
DD 1	29,947,800	18,808,543 (62.80%)	98.72%	93.50%	1,428,828
DD 2	24,083,779	216,430,52 (89.87%)	98.94%	94.67%	426,634
DD 3	48,583,019	45,634,320 (93.93%)	98.91%	94.62%	1,468,324
DD 4	47,625,147	41,735,829 (87.63%)	98.91%	94.02%	12,845,203
DD 5	50,936,612	46,158,268 (90.62%)	98.69%	93.32%	7,900,045
DD 6	28,051,208	16,648,215 (59.35%)	98.81%	94.20%	1,448,967
DD 7	81,098,971	67,786,634 (83.59%)	98.98%	95.11%	2,177,393
DD 8	50,374,765	44,617,225 (88.57%)	98.91%	93.87%	1,746,933
DD 9	54,017,791	51,321,862 (95.01%)	99.12%	97.56%	4,259,498
DD 10	38,095,238	31,881,721 (83.69%)	98.64%	96.35%	7,543,771
Control 1	55,492,708	47,926,094 (86.36%)	99.06%	94.58%	681,164
Control 2	50,594,530	46,373,575 (91.66%)	98.83%	93.58%	3,380,139
Control 3	37,387,520	25,946,219 (69.40%)	98.91%	94.44%	856,419
Control 4	44,852,156	37,645,238 (83.93%)	98.98%	94.14%	1,284,731
Control 5	20,785,656	45,634,320 (93.93%)	98.91%	94.48%	234,324
Control 6	38,977,001	35,108,194 (90.07%)	98.89%	97.31%	2,494,358
Control 7	25,745,004	19,298,221 (74.96%)	99.04%	94.28%	299,844
Control 8	33,928,731	30,275,015 (89.23%)	98.99%	93.80%	6,523,253
Control 9	55,606,502	49,864,823 (89.67%)	99.12%	94.07%	3,003,336
Control 10	33,322,179	27,434,382 (82.33%)	99.01%	94.17%	399,765

Q20: The percentage of base calling data with accuracy of more than 99% during high-throughput sequencing. Q30: The percentage of base calling data with accuracy of more than 99.9% during high-throughput sequencing.

**Table 3:** Dysregulated miRNAs in patients with lumbar disc herniation (Disc Degeneration Group) versus the Control Group.

Upregulated miRNA	log2 Fold Change	*p*-Value	*p*-adj
hsa-miR-4685-3p	4.3478	7.24 × 10^−7^	0.000241
hsa-miR-766-3p	3.0624	4.42 × 10^−6^	0.000458
hsa-miR-3605-3p	3.1164	7.75 × 10^−6^	0.000574
hsa-miR-6749-3p	4.667	0.000152	0.005617
hsa-miR-1227-3p	4.4573	0.0002	0.00666
hsa-miR-6726-3p	6.0323	0.000518	0.011509
hsa-miR-877-3p	4.2815	0.000493	0.011509
hsa-miR-197-3p	2.5895	0.000699	0.013684
hsa-miR-6819-3p	2.9766	0.001757	0.02489
hsa-miR-3620-3p	4.9605	0.00228	0.028649
hsa-miR-1908-3p	3.0883	0.002361	0.029119
hsa-miR-211-5p	4.4374	0.002771	0.03296
hsa-miR-342-3p	2.0702	0.003209	0.034472
hsa-miR-130b-5p	2.2154	0.003278	0.034655
hsa-miR-654-5p	2.223	0.003642	0.037897
hsa-miR-6894-3p	4.6656	0.004032	0.03892
hsa-miR-543	3.4698	0.005086	0.046401
**Downregulated miRNA**	**log2 Fold Change**	***p*-Value**	***p*-adj**
hsa-miR-4452	−7.819	2.07 × 10^−7^	0.000138
hsa-miR-1-3p	−4.3578	1.81 × 10^−6^	0.000304
hsa-miR-5006-5p	−6.8699	1.83 × 10^−6^	0.000304
hsa-miR-6165	−9.193	2.44 × 10^−6^	0.000325
hsa-miR-449a	−9.3266	5.5 × 10^−6^	0.000458
hsa-miR-4632-5p	−7.6058	4.86 × 10^−6^	0.000458
hsa-miR-1303	−4.4186	3.37 × 10^−5^	0.002007
hsa-miR-219a-2-3p	−5.8958	3.31 × 10^−5^	0.002007
hsa-miR-4301	−5.618	3.62 × 10^−5^	0.002007
hsa-miR-27a-3p	−3.0627	5.02 × 10^−5^	0.002572
hsa-miR-15b-5p	−3.7083	6.13 × 10^−5^	0.002722
hsa-miR-566	−6.0829	5.77 × 10^−5^	0.002722
hsa-miR-5096	−2.8231	9.08 × 10^−5^	0.003781
hsa-miR-1285-5p	−3.0624	0.000109	0.004272
hsa-miR-6859-3p	−5.8857	0.000181	0.006354
hsa-miR-181c-3p	−5.4625	0.000238	0.006678
hsa-miR-200b-3p	−4.8407	0.000211	0.006678
hsa-miR-320c	−2.6456	0.000241	0.006678
hsa-miR-4762-3p	−6.4726	0.000228	0.006678
hsa-miR-5197-5p	−7.0974	0.000325	0.008651
hsa-miR-320d	−3.0007	0.000338	0.008669
hsa-miR-7847-3p	−5.6968	0.000396	0.009769
hsa-miR-4261	−6.8466	0.000514	0.011509
hsa-miR-125b-5p	−3.7142	0.000542	0.011643
hsa-miR-1290	−2.4541	0.000669	0.013494
hsa-miR-4488	−4.5272	0.000667	0.013494
hsa-miR-762	−5.9577	0.00074	0.014083
hsa-miR-5591-3p	−7.439	0.00087	0.015669
hsa-miR-5591-5p	−7.439	0.00087	0.015669
hsa-miR-7704	−3.2961	0.000975	0.017088
hsa-miR-548ba	−6.598	0.001137	0.018927
hsa-miR-548d-5p	−6.3677	0.001123	0.018927
hsa-miR-99a-5p	−1.7454	0.001213	0.019711
hsa-miR-3178	−4.214	0.001295	0.020535
hsa-miR-29a-3p	−3.5301	0.001448	0.02242
hsa-miR-1273c	−3.6329	0.001542	0.023337
hsa-miR-195-5p	−3.8204	0.001641	0.024281
hsa-miR-16-5p	−1.9705	0.001687	0.024423
hsa-miR-1246	−2.304	0.002231	0.028649
hsa-miR-320b	−2.0358	0.002273	0.028649
hsa-miR-3653-3p	−4.9585	0.002241	0.028649
hsa-miR-4721	−7.0703	0.002176	0.028649
hsa-miR-6849-3p	−6.8278	0.002274	0.028649
hsa-miR-1181	−4.7855	0.002671	0.03234
hsa-miR-141-3p	−3.2258	0.003021	0.034104
hsa-miR-26b-5p	−2.6011	0.002971	0.034104
hsa-miR-616-3p	−3.7777	0.002987	0.034104
hsa-miR-199a-3p	−1.9663	0.003127	0.03414
hsa-miR-6728-3p	−6.8316	0.003115	0.03414
hsa-miR-4484	−6.6985	0.003787	0.038356
hsa-miR-450a-5p	−3.6107	0.003801	0.038356
hsa-miR-143-3p	−2.002	0.004012	0.03892
hsa-miR-4516	−4.2535	0.003987	0.03892
hsa-miR-144-5p	−5.0596	0.00481	0.045767
hsa-miR-340-5p	−5.3597	0.00503	0.046401
hsa-miR-5010-5p	−3.1516	0.005051	0.046401

Padj is the *p* value adjusted by multiple hypothesis testing correction (Benjamini and Hochberg’s method). Padj is also known as FDR (false discovery rate) or Q value.

**Table 4:** Intervertebral disc (IVD) degeneration-related miRNA-target gene pathway regulation.

Pathway	miRNAs Upregulated		Target Genes	miRNAs Downregulated		Target Genes
endocytosis	miR-766-3p	miR-4685-3p	ARAP1, ZFYVE27, HSPA6, PSD4, ARF1, VPS37C, TGFBR1, IGF1R, PSD3, CBL	miR-4632-5p	miR-5006-5p	MARC1
apoptosis	miR-766-3p	miR-6749-3p	CTSK, RIPK1, HRK, ERN1, CASP10, PIK3CD, ATM, TRAF2, MAPK1, TRAF1	miR-4632-5p	miR-6165	PDPK1, NRAS, RELA, CASP2, PIK3CB, CASP10, AKT2, HRK, TRAF2, XIAP
axon guidance	miR-766-3p	miR-6749-3p	Clorf220, SLIT3, SSH3, WNT4, HRAS, LIMK1, ENAH, C15orf37, PIK3R1, NTN1	miR-4632-5p	miR-5006-5p	NRAS, PAK4, SEMA4F, RYK, PIK3CB, PLXNA3, ABL1, PAK3, SEMA4A, PLCG1
VEGF	miR-766-3p	miR-6749-3p	Clorf220, MAPK13, HRAS, C15orf37, PIK3R1, MAPK14, PLCG1, SHPK, CDC42, PRKCA	miR-4632-5p	miR-1303	MAPKAPK2, NRAS, AKT2, PPP3CA, PLCG2, PIK3R3, MAPK1, MAPK13, AKT1, MARC1
regulation of cytoskeleton	miR-766-3p	miR-6749-3p	Clorf220, SSH3, HRAS, ARHGEF7, ITGA8, LIMK1, ENAH, ITGA11, C15orf37, ITGAX	miR-4452	miR-1303	COLGA6L9, MARCH1, MRC1
cell adhesion molecular	miR-766-3p	miR-6749-3p	Clorf220, CD226, F11R, ICOS, ITGA8, C15orf37, TIGIT, NRXN2, JAM2, SDC4	miR-4632-5p	miR-5006-5p	F11R, MPZ, PVRL1, SDC3, CLDN18, SDC2, NFASC, MPZL1, SPN, ITGA9
focal adhesion	miR-766-3p	miR-6749-3p	Clorf220, HRAS, IGF1, ITGA8, TLN2, ITGA11, TNR, C15orf37, PIK3R1, LAMC2	miR-4632-5p	miR-6165	IGF1R, PDGFRB, PDPK1, BAB2, VCL, PAK4, PARVA, PIK3CB, VEGFA, ITGA9
ECM-receptor interaction	miR-766-3p	miR-6749-3p	Clorf220, DAG1, ITGA8, ITGA11, TNR, C15orf37, LAMC2, SV2B, SDC4, SHPK	miR-4632-5p	miR-5006-5p	COL6A1, TNR, COL4A4, ITGA1, ITGA3, LAMC3, ITGA8, ITGA9, Sv2c
PI3K-AKT	miR-766-3p	miR-6749-3p	Clorf220, HRAS, IGF1, JAK3, ITGA8, INSR, GNB4, GNG4, ITGA11, TNR	miR-4632-5p	miR-1303	FGF23, NRAS, RELA, CDKN1B, CCNE2, PPP2R5C, FGF14, ITGA9, COL4A4, AKT2
mTOR	miR-766-3p	miR-6749-3p	Clorf220, WNT9B, WNT4, HRAS, IGF1, WNT2B, INSR, C15orf37, PIK3R1, GRB2	miR-5006-5p	miR-6165	WNT2B, PDPK1, PIK3CB, MAPK1, FZD8, GOLGA6L9, WNT7B, RPS6KA2, NPRL3, WNT3
NF-Kb	miR-766-3p	miR-6749-3p	PLCG1, TMEM236	miR-4632-5p	miR-5006-5p	MYD88, TNFRSF13C, TRIM25, RELA, IL1R1, TNFAIP3, TNFRSF11A, TRAF3, PLCG2, TRAF1

## Data Availability

The dataset generated and analyzed during the current study is openly available under the BioProject accession number PRJNA657132.
